# FoxO1 Gain of Function in the Pancreas Causes Glucose Intolerance, Polycystic Pancreas, and Islet Hypervascularization

**DOI:** 10.1371/journal.pone.0032249

**Published:** 2012-02-23

**Authors:** Osamu Kikuchi, Masaki Kobayashi, Kosuke Amano, Tsutomu Sasaki, Tomoya Kitazumi, Hye-Jin Kim, Yong-Soo Lee, Hiromi Yokota-Hashimoto, Yukari-Ido Kitamura, Tadahiro Kitamura

**Affiliations:** Metabolic Signal Research Center, Institute for Molecular and Cellular Regulation, Gunma University, Maebashi, Gunma, Japan; University of Bremen, Germany

## Abstract

Genetic studies revealed that the ablation of insulin/IGF-1 signaling in the pancreas causes diabetes. FoxO1 is a downstream transcription factor of insulin/IGF-1 signaling. We previously reported that FoxO1 haploinsufficiency restored β cell mass and rescued diabetes in IRS2 knockout mice. However, it is still unclear whether FoxO1 dysregulation in the pancreas could be the cause of diabetes. To test this hypothesis, we generated transgenic mice overexpressing constitutively active FoxO1 specifically in the pancreas (TG). TG mice had impaired glucose tolerance and some of them indeed developed diabetes due to the reduction of β cell mass, which is associated with decreased Pdx1 and MafA in β cells. We also observed increased proliferation of pancreatic duct epithelial cells in TG mice and some mice developed a polycystic pancreas as they aged. Furthermore, TG mice exhibited islet hypervascularities due to increased VEGF-A expression in β cells. We found FoxO1 binds to the VEGF-A promoter and regulates VEGF-A transcription in β cells. We propose that dysregulation of FoxO1 activity in the pancreas could account for the development of diabetes and pancreatic cysts.

## Introduction

Pancreatic β cells secrete insulin to maintain plasma glucose levels at an appropriate physiological range. Relative defects in β cell functions cause type 2 diabetes. Recent genetic studies revealed that insulin/IGF-1 signaling plays a role in β cell growth and function [1], [2]. The insulin/IGF-1 signaling pathway in β cells is mainly mediated by insulin receptor substrate-2 (IRS-2), PI3-kinase, 3-phosphoinositide-dependent protein kinase 1 (Pdk-1), and Akt. Mice lacking IRS-2 develop diabetes due to reduced β cell mass and peripheral insulin resistance [3], [4]. Mice lacking Pdk-1, specifically in pancreatic β cells, develop progressive hyperglycemia ensued from a loss of islet mass [5]. Transgenic mice overexpressing the active form of Akt1 under the rat insulin promoter had increased numbers of β cells and high plasma insulin levels, leading to improved glucose tolerance and resistance to diabetes [6].

The FoxO (Forkhead box-containing protein, O-subfamily) transcription factors are downstream effectors of insulin/IGF-1 signaling. Insulin/IGF-1 activates PI3-kinase/Akt pathway. Activated Akt translocates to the nucleus and phosphorylates FoxO1, which leads from nucleus to cytoplasm translocation of FoxO1. Because FoxO1 is inactive in the cytoplasm, insulin/IGF-1 pathway essentially inhibits FoxO1 transcriptional activity [7], [8], [9]. The FoxO family contains four isoforms, FoxO1, FoxO3a, FoxO4, and FoxO6; FoxO1 is the most abundant isoform in pancreatic β cells [10]. Haploinsufficiency for FoxO1 resulted in an increase of β cells and rescued both IRS-2 knockout mice and Pdk-1 knockout mice from diabetes via restoration of Pdx1 expression in β cells [5], [10]. Pdx1 is a key transcription factor for β cell growth and function [11], [12]. *In vitro* assays in β cell cultures revealed that FoxO1 inhibits Pdx1 transcription by competing with FoxA2 for a common binding site in the Pdx1 promoter [10]. FoxO1 and Pdx1 have been reported to show mutually exclusive nuclear localization [5], [13], [14]. Interestingly, the expression pattern of FoxO1 during mouse pancreas development closely parallels Pdx1 expression, i.e. widely expresses at E14.5, becomes restricted to endocrine cells at E17.5, and is confined to β cells postnatally; the difference is that FoxO1 is cytoplasmic and Pdx1 nuclear [15]. On the other hand, we also reported that FoxO1 controls myogenic differentiation cooperatively with Notch signaling [16]. Notch signaling is critical for pancreatic cell and myogenic differentiation [17], [18]. Thus, the accumulated evidence suggests FoxO1 dysregulation in the pancreas could be the cause of diabetes or pancreatic disease.

To test this hypothesis *in vivo*, we generated pancreas specific FoxO1 transgenic mice (TG) [15]. TG mice constitutively express the nuclear FoxO1 mutant (FoxO1-ADA) [19] under the Pdx1 promoter [17], and have transgene expression in >90% of pancreatic cells [15]. TG mice have reduced exocrine acinar cells and abnormal composition of islet cells during pancreas development [15]. However, glucose metabolism and pancreatic architecture in adult TG mice have not been characterized yet. Therefore, in the present study, we investigated metabolism parameters and pancreas morphology in adult TG mice. We showed that dysregulation of FoxO1 in pancreas causes diabetes, polycystic pancreas and islet hypervascularization.

## Materials and Methods

### Animal generation and analytical procedures

Pdx-FoxO1ADA transgenic mice generated by a transgene encoding the 4.5 kb Pdx1 promoter [17] followed by the cDNA of FLAG-tagged FoxO1ADA [19] were described previously [15]. PCR genotyping was carried out with the following primers: 5′-GCT TAG AGC AGA GAT GTT CTC ACA TT-3′, 5′-CCA GAG TCT TTG TAT CAG GCA AAT AA-3′, and 5′-CAA GTC CAT TAA TTC AGC ACA TTG A-3′. Individually caged mice were fed either normal chow or high fat high sucrose diet (HFHSD) beginning at 4 weeks of age for an 8-week period. In the HFHSD, 55% of calories were from fat, 28% from carbohydrates, and 17% from protein (Oriental Yeast Co., Ltd). We purchased male *db/db* mice from CLEA Japan (Tokyo, Japan). All animal care and experimental procedures were approved by the Institutional Animal Care and Use Committee at Gunma University (#06-54 and #08-01). All animal experimentation described in the manuscript was conducted in accordance with accepted standards of humane animal care, as outlined in the ethical guidelines. We measured blood glucose levels with a glucometer (Sanwa Kagaku, Nagoya), and plasma insulin levels by ELISA (Shibayagi) and plasma glucagon levels by RIA (Millipore). We carried out all assays in duplicate. Each value represents the mean of two independent determinations. For the glucose tolerance test, we subjected mice to an overnight fast followed by an intraperitoneal glucose injection (1.2 g/kg), and obtained blood samples 0, 15, 30, 60, and 120 min after the injection. For insulin tolerance test, we injected human insulin (0.75 U/kg) intraperitoneally and obtained blood samples 0, 15, 30 and 60 min after the injection.

### Antibodies and immunohistochemistry

We used the following antibodies: anti-Pdx1 (a kind gift from Dr Kaneto at Osaka Univ), anti-insulin (DAKO), anti-glucagon (Sigma), anti-FLAG (Sigma), anti-Ki67 (Lab Vision), anti-MafA (Bethyl), anti-PECAM1 (Endogen), and anti-VEGF-A (Fitzgerald). We used fluorescent-conjugated DBA (0.05 mg/ml, Vector Laboratories) for duct epithelial cell staining. We performed immunostaining using 5 µm-thick paraffin sections and, in some experiments, antigen retrieval, as described previously [10]. We visualized immune complexes with FITC- or CY3-conjugated secondary antibodies. To quantify the % area of β cells or α cells vs. pancreas, we measured the area of insulin staining or glucagon staining using BZ-8100 (Keyence) and NIS-Elements (Nikon). We scored at least 10 sections for each mouse and six mice for each genotype.

### Islet isolation from mice

Islets were purified from mice by collagenase digestion followed by centrifugation over a Histopaque gradient, as described previously [20].

### Cell culture and adenoviral vectors

βTC3 and MIN6 cells were kindly provided from D. Accili (Columbia University) [10]. We previously described adenoviral vectors encoding FoxO1-ADA or GFP [19]. We infected the cells with adenovirus 24 hrs prior to isolating mRNA or to preparing samples for luciferase assays.

### Luciferase assays and chromatin immunoprecipitation (ChIP) aasays

We performed luciferase assays as previously described [10]. We generated a VEGF-A luciferase vector containing the promoter region (−1217 to +180) of mouse VEGF-A (pGL3-VEGF-A). We performed ChIP assays in MIN6 cells following previously described methods [21]. We used anti-FoxO1 (Santa-Cruz, H-128), anti-FoxO3a (Santa-Cruz, H-144) antibodies and the following primers: FHRE5; 5′-CTT CCC AGA GGA TCC CAT TCA CCC C-3′ and FHRE3; 5′-GCC GAG CGC CCC CTA GTG AC-3′ (corresponding to −805 to −781 and −525 to −506 of the mouse VEGF-A gene, respectively). As control primers, we used Cont5; 5′-TCC CAG TGT GTT CCT GAG CCC A-3′ and Cont3; 5′-TCT CGC GAC AGA GCT CCG CT-3′ (corresponding to −220 to −199 and +68 to +87 of the mouse VEGF-A gene, respectively).

### mRNA isolation and real-time RT-PCR

We extracted mRNA from mouse islets, β TC3, or MIN6 cells using the Micro Fast Track 2.0 kit (Invitrogen). We performed real-time RT-PCR using the ImProm-II™ Reverse Transcription System (Promega) and LightCycler System (Roche). Primer sequences used for real-time PCR are as follows, for PECAM1; 5′- AGG GGA CCA GCT GCA CAT TAG G-3′ and 5′-AGG CCG CTT CTC TTG ACC ACT T-3′, for VEGF-A; 5′- TGT ACC TCC ACC ATG CCA AGT-3′ and 5′- TGG AAG ATG TCC ACC AGG GT-3′, for Pdx1; 5′- ACC ATG AAC AGT GAG GAG CA -3′ and 5′- TCC TCT TGT TTT CCT CGG GT-3′, for MafA; 5′- AGG CCT TCC GGG GTC AGA G -3′ and 5′- TGG AGC TGG CAC TTC TCG CT-3′. We carried out each reaction in triplicate, using a standard curve with the relevant cDNA for each primer set.

### Statistical analyses

We carried out descriptive statistics and analysis of variance (ANOVA) followed by Fisher's test using the Statview software (Abacus concepts).

## Results

### Hyperglycemia and impaired glucose tolerance in TG mice

**Figure 1:**
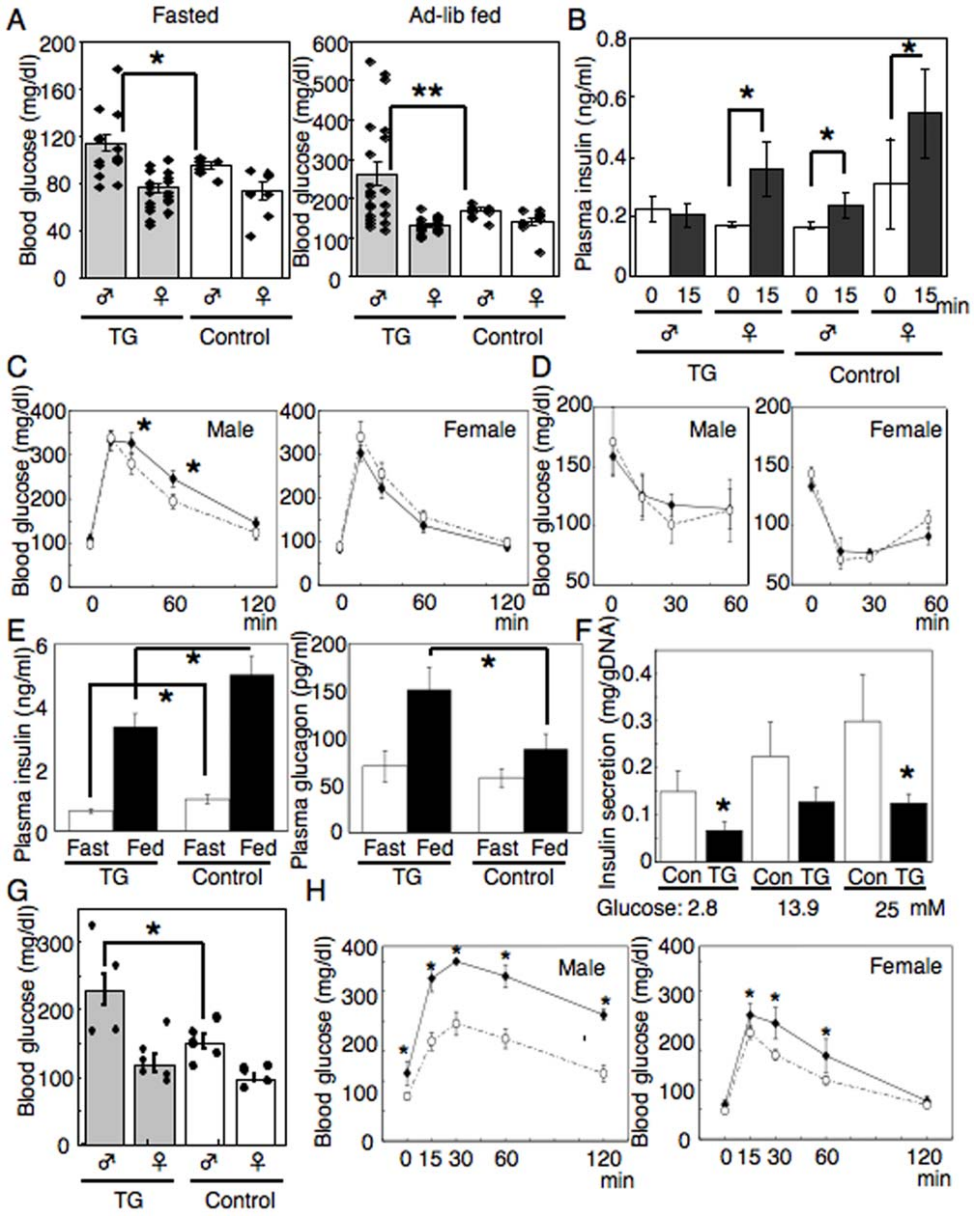
Hyperglycemia and impaired glucose tolerance in TG mice. (A) Blood glucose levels (mg/dl) in fasted and ad-lib fed TG mice and their control littermates at 8 weeks of age. (B) Plasma insulin levels (ng/ml) before and 15 min after glucose (1.2 g/kg) intraperitoneal injection. (C) Intraperitoneal glucose tolerance tests (IPGTT) in male (left) and female (right) TG mice that had normal blood glucose levels in fasted and fed states at 8 weeks of age. (D) Insulin tolerance test (ITT) in male (left) and female (right) TG and control mice at 12 weeks of age. (E) Plasma insulin (left) and glucagon (right) levels in fasted and fed conditions in TG and control mice at 12 weeks of age. (F) The size-matched islets were isolated from male TG and control mice at 12 weeks of age, and glucose-stimulated insulin secretion levels were measured in those islets. The data were normalized by DNA content in those islets (G and H) TG mice that had normal blood glucose levels at 4 weeks of age and their control littermates were fed a HFHSD for 8 weeks further, then, fasted blood glucose levels were measured (G) and IPGTT performed (H) in both male (left) and female (right) mice. In each experiment (A–H), at least six male or female mice for each genotype were analyzed. In the line graphs, closed triangle indicates TG mice and open circle indicates control mice. Data represent mean ± SEM. * or ** indicates *P*<0.05 or *P*<0.01 by ANOVA.

**Figure 2:**
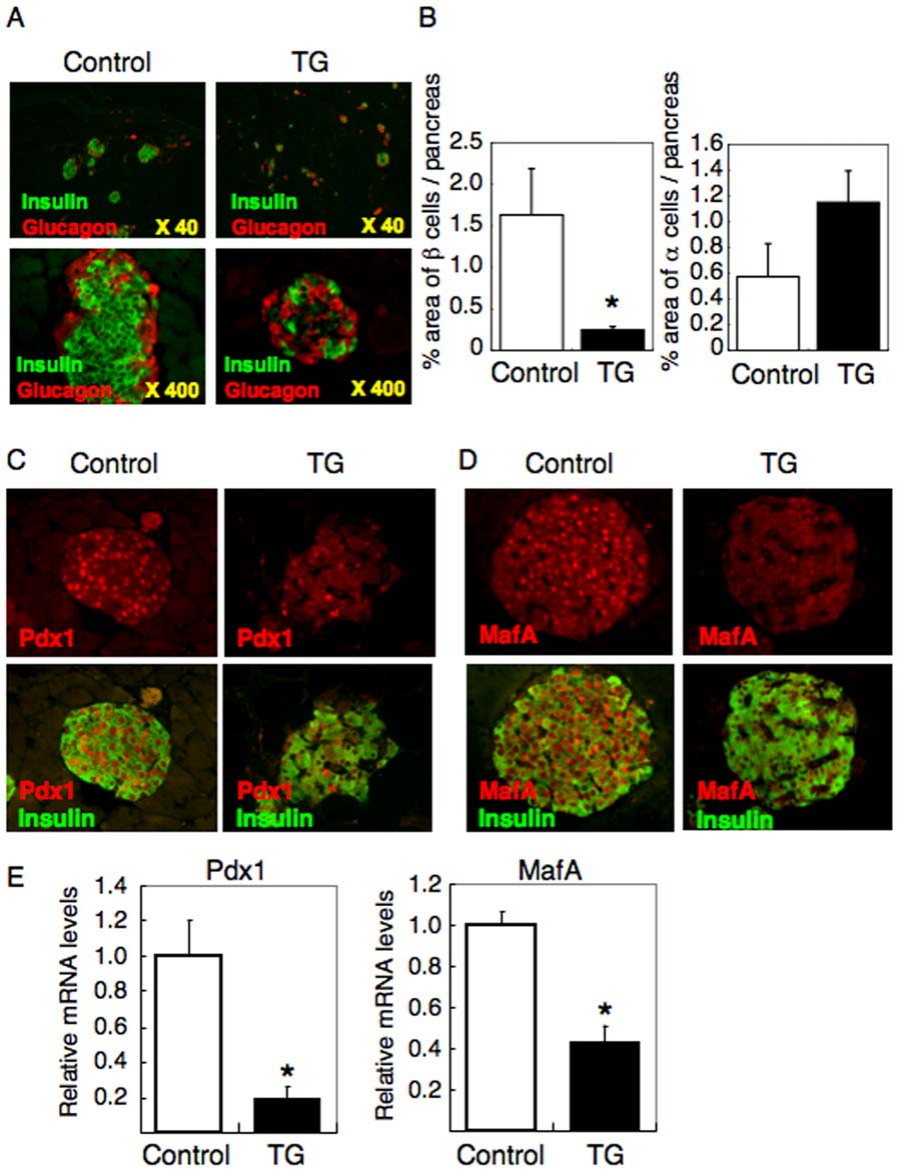
Reduced β cell mass and decreased expression of Pdx1 and MafA in TG mice. (A) Double immunohistochemistry with anti-insulin and anti-glucagon antibodies were conducted in pancreatic sections from TG and control mice. Representative images of low (upper panels) and high (lower panels) magnifications are shown. (B) The % area of β cells (left panel) or α cells (right panel) vs. whole pancreatic area was scored as described in Methods. (C and D) Double immunostaining of insulin with Pdx1 (C) or MafA (D) were conducted in pancreatic sections from TG and control mice. Arrows indicate individual cysts in TG pancreas. Representative images are shown. (E) Islets were isolated from TG and control mice and used for analyses by real time RT-PCR. In each experiment (A–E), at least six male and six female mice of each genotype and ten sections per mouse were analyzed. All the results were normalized using GAPDH. There was no significant difference between male and female mice. Data represent mean ± SEM. An asterisk indicates *P*<0.05 by ANOVA.

We generated transgenic mice overexpressing constitutively active FoxO1 specifically in the pancreas, using a transgene encoding a 4.5 kb Pdx1 promoter [17] followed by the cDNA of a constitutively nuclear mutant of FoxO1 (FoxO1-ADA) [19]. In this system, the Pdx1 promoter could be inhibited by its FoxO1 transgene product, however we confirmed that more than 90% of pancreatic cells expressed FoxO1-ADA in these mice [15]. One possibility is that because Pdx1 promoter is in fact inhibited by FoxO1-ADA, the level of transgene expression is only twofold higher than endogenous gene [15]. Another possibility is that the inhibition of endogenous Pdx1 expression is not necessarily a result of a direct effect of FoxO1, but it can be secondary to increased mRNA degradation or to promoter elements located outside the region we used to make transgenic mouse. Though Pdx1 is known to express in the brain as well as pancreas, FoxO1-ADA expression was observed neither in the brain nor in the other organs except pancreas of TG mice (data not shown). Although we already reported the phenotype of pancreatic morphology during pancreas development in TG mice [15], the other phenotypes in adult TG mice, such as metabolic parameters and pancreas architecture remained unsolved. Therefore, we first examined blood glucose levels at 8 weeks of age from both fasted and ad-lib fed mice. As seen in Fig. 1A, around 30% of male TG mice showed hyperglycemia in both fasted and fed conditions. Interestingly, none of the female TG mice developed hyperglycemia. Plasma insulin secretion in response to intraperitoneal glucose injection was blunted in male diabetic TG mice, indicating that hyperglycemia was caused by impaired insulin secretion in these mice (Fig. 1B). However, around 70% of male and all female TG mice showed almost normal blood glucose levels (Fig. 1A); therefore, we next performed intraperitoneal glucose tolerance tests (IPGTT) in the TG mice that had normal blood glucose levels in fasted and fed states at 12 weeks of age. IPGTT revealed that male, but not female, TG mice had impaired glucose tolerance; although, statistical significance was seen only at 30 and 60 min (Fig. 1C). There was no difference in the average body weight between TG and control mice (TG; 24.3±1.1 g, control; 24.5±1.4 g). To rule out the possibility of insulin resistance in TG mice, we performed insulin tolerance test (ITT). There was no difference in ITT results between TG and control mice in both male and female (Fig. 1D), indicating that impaired glucose tolerance was not caused by the peripheral insulin resistance in TG mice. We also measured plasma insulin and glucagon levels in male TG mice. As shown in Fig. 1E, plasma insulin levels were significantly lower in TG than control mice in both fasted and fed conditions, which is consistent with the impaired glucose responsive insulin secretion in male TG mice (Fig. 1B). Interestingly, plasma glucagon levels were higher in TG than control mice in the fed conditions (Fig. 1E). Although the mechanism is unclear, enhanced glucagon secretion may deteriorate the glucose intolerance in TG mice. To further evaluate the impaired insulin secretion in TG mice, we isolated islets from TG and control mice and performed glucose stimulated insulin secretion (GSIS) assays. Because small islets in TG mice have lower β cell/α cell ratio than in the control mice (as shown in Fig. 2A), we used the size-matched large islets between TG and control mice, in which β cell mass should be comparable. Consistent with the data of plasma insulin levels, TG islets have decreased glucose-responsive insulin secretion (Fig. 1F). We next started feeding a high fat high sucrose diet (HFHSD) to the TG mice whose blood glucose levels were normal, at least until 4 weeks of age. After HFHSD feeding for 8 weeks, we measured blood glucose levels and found the average blood glucose level in male TG mice was significantly higher than in control mice, and these mice developed overt diabetes (Fig. 1G). IPGTT revealed that not only male, but also female TG mice exhibited significantly impaired glucose tolerance under a HFHSD (Fig. 1H).

### Reduced β cell mass and decreased expression levels of Pdx1 and MafA in TG mice

We previously showed FoxO1 inhibits β cell proliferation through suppression of Pdx1 transcription in β cells [10]; therefore, we expected β cell mass should be decreased in TG mice. Double staining of insulin and glucagon in pancreatic sections revealed the β cell area decreased while the α cell area was relatively increased in TG mice compared to control mice (Fig. 2A). We quantified the area of β cells or α cells as a % of the whole pancreatic area, showing that the β cell area was significantly decreased, whereas α cell area tended to be increased in TG mice compared to control mice (Fig. 2B). Although abnormalities in glucose metabolism were milder in female than male TG mice, we couldn't see any difference in pancreas morphology between males and females. We next investigated Pdx1 expression in the pancreas using immunohistochemistry with anti-Pdx1 antibody. For this investigation, we selected the TG islets which contain the similar number of β cells to the control islets. In control mice, Pdx1 is expressed in almost all insulin-positive β cells, whereas only a few insulin-positive β cells express Pdx1 in TG mice (Fig. 2C). These results are consistent with our previous hypothesis that FoxO1 inhibits Pdx1 transcription and, thereby, inhibits β cell proliferation [10]. Although we have also reported that FoxO1 is a positive regulator for MafA transcription [21], MafA expression was decreased in β cells of TG mice (Fig. 2D). To quantify these results, we isolated islets from TG and control mice and performed real time RT-PCR analysis. Consistent with the results of immunohistochemistry, both Pdx1 and MafA expression levels were significantly decreased in TG mice (Fig. 2E). However, given that the % of β cells in the islets is decreased in TG mice and that Pdx1 and MafA are exclusively expressed in β cells, these quantitative data may overestimate the reduction of Pdx1 and MafA expression. Nonetheless, these combined results of immunohistochemistry and real time RT-PCR indicated that Pdx1 and MafA, two critical transcription factors for β cell proliferation, are decreased, which may be the cause of decreased β cell mass in TG mice.

### Enhanced proliferation of pancreatic duct epithelial cells and development of polycystic pancreas in TG mice

**Figure 3:**
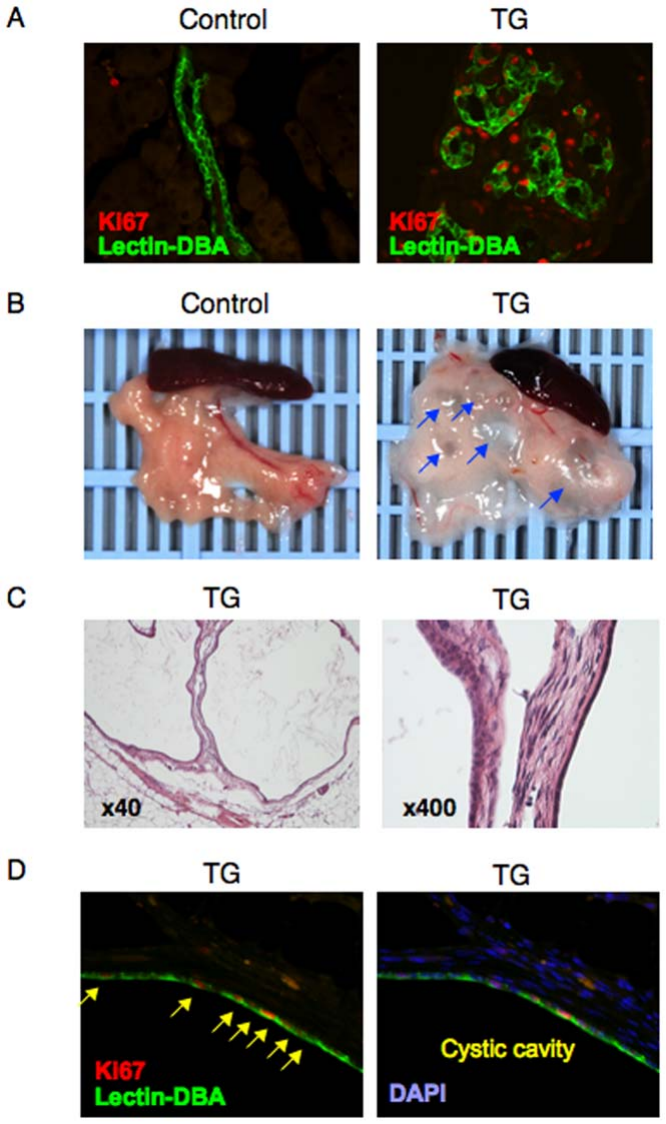
Enhanced proliferation of pancreatic duct epithelial cells and development of polycystic pancreas in TG mice. (A) Immunohistochemistry with anti-Ki67 antibody (red) along with Lectin-DBA (green) was conducted in pancreatic sections from 2 month old TG and control mice. Representative results are shown. (B) Photographs of the pancreas in 12 month old TG and control mice. Representative pictures are shown. (C) Hematoxylin and eosin (HE) staining (left panel indicates ×40 magnification and right ×400) in pancreatic sections from 12 month old TG mice. Representative images are shown. (D) Immunohistochemistry with anti-Ki67 antibody (red) along with Lectin-DBA (green) was conducted in pancreatic sections from 12 month old TG mice. Representative images of pancreatic cysts are shown. Arrow indicates Ki67 positive cells.

As we previously reported [15], the TG pancreas contained numerous duct-like structures. Lectin-DBA staining, a marker for duct epithelial cells, revealed that these structures were indeed pancreatic ducts (Fig. 3A). More interestingly, most of the Lectin-DBA positive cells in the TG pancreas were Ki67 positive, indicating that duct cells were vigorously proliferating in these mice (Fig. 3A, right panel). The turnover of pancreatic duct epithelial cells is essentially very slow, as seen in pancreatic sections from control mice where no Ki67 positive cells were detected in the duct (Fig. 3A, left panel). As a consequence of vigorous and continuous proliferation of pancreatic duct epithelial cells, we expected TG mice might develop pancreatic cysts in their old age due to the formation of enlarged pancreatic ducts. As expected, multiple cysts formed in the pancreas of 12 month old TG mice (Fig. 3B). According to the cytopathological malignant criteria, pancreatic cysts in TG mice contained no malignant cells, indicating these cysts were not cancerous (Fig. 3C). Furthermore, pancreatic cysts were formed by a monolayer of Lectin-DBA positive duct epithelial cells, most of which were Ki67 positive (Fig. 3D).

### Islet hypervascularities in TG mice are associated with increased VEGF-A expression in β cells

**Figure 4:**
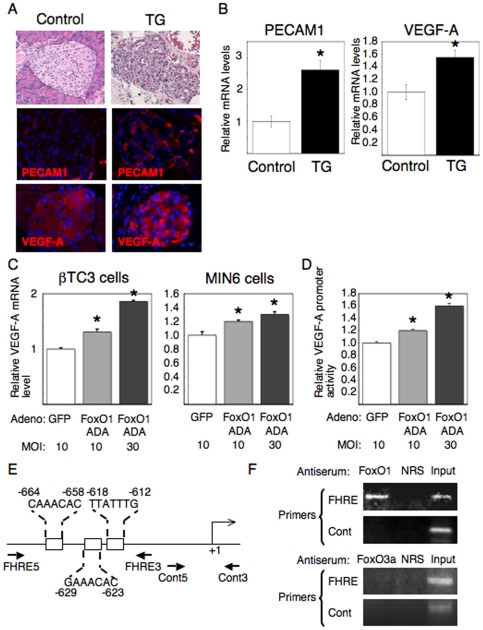
Islet vasculature and VEGF-A expression in β cells are increased in TG mice via FoxO1 regulation of VEGF-A transcription. (A) Hematoxylin and eosin (HE) staining and immunohistochemistry with anti-PECAM1 or anti-VEGF-A antibodies in pancreatic sections from 2 month old TG and control mice. Representative images are shown. (B) Islets were isolated from TG and control mice and used for analyses by real-time RT-PCR to quantify PECAM1 and VEGF-A mRNA levels. In each experiment (A and B), six male and six female mice were analyzed for each genotype. There was no difference between male and female mice. Data represent the mean ± SEM fold-increase relative to control mice. An asterisk indicates *P*<0.05 by ANOVA. (C) β TC3 or MIN6 cells were infected with adenoviruses expressing FoxO1 ADA or GFP at indicated MOI, 24 hrs later mRNA was isolated from the cells and used for real-time RT-PCR to quantify VEGF-A mRNA levels. The results of (B) and (C) were normalized using GAPDH. (D) VEGF-A promoter driven luciferase activities were measured in MIN6 cells infected with adenoviruses expressing FoxO1 ADA or GFP at indicated MOI. For (C) and (D), data represent the mean ± SEM fold-increase relative to GFP infection. An asterisk indicates *P*<0.05 by ANOVA. (E) The promoter region of the mouse VEGF-A gene. Square indicates the location of the forkhead responsive element (FHRE). For ChIP assays, a primer set spanning three FHREs and control primers were used. An arrow indicates the initiation site of RNA synthesis and is designated +1. (F) ChIP assays of the VEGF-A promoter are shown. We performed ChIP assays in MIN6 cells with indicated antisera. NRS indicates normal rabbit serum used as a control antisera. The crosslinked DNA was amplified by PCR using FHRE and Cont primers. Input represents DNA extracted from chromatin prior to immunoprecipitation. We used input DNA with 1∶100 dilution.

**Figure 5:**
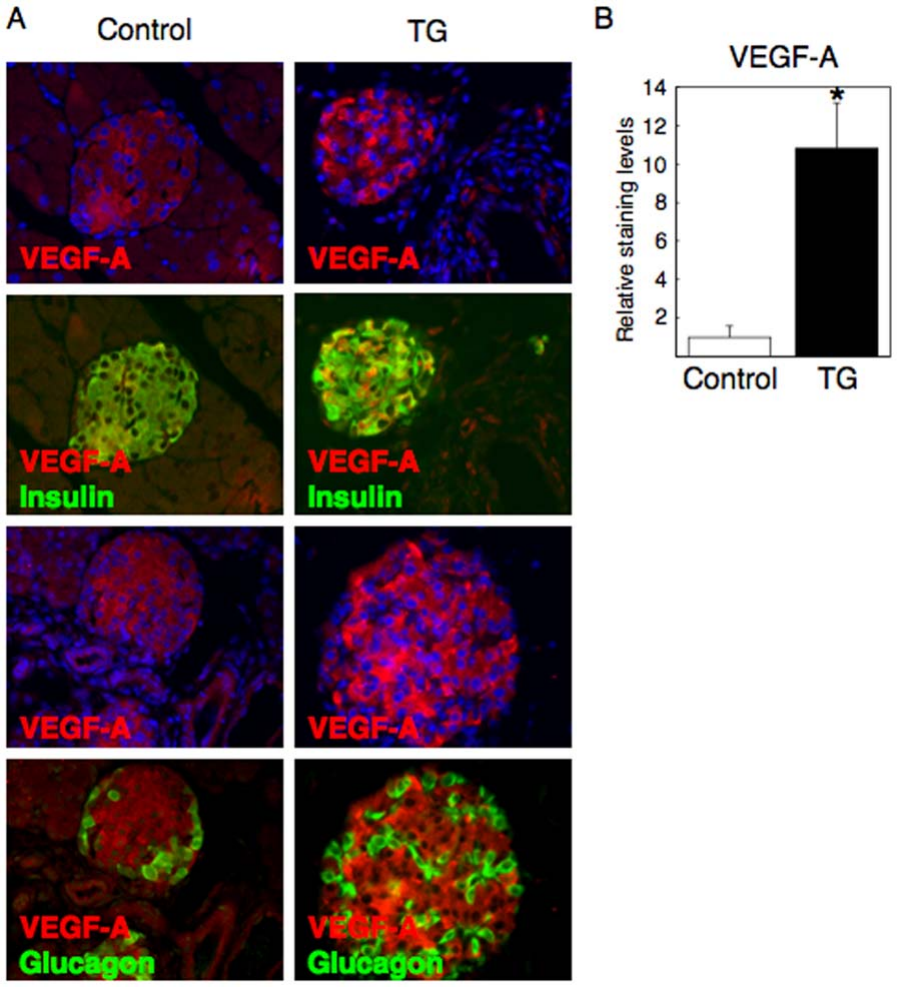
Enhanced VEGF-A expression in β cells but not α cells in TG mice. (**A**) Double immunostaining of VEGF-A with insulin or glucagon in pancreatic sections from TG mice and control mice. Enhanced VEGF-A staining (red) in TG mice is completely merged with insulin staining (green to yellow) but not with glucagon staining (green). (B) Quantitative analysis of VEGF-A staining levels in the islets of TG and control mice. We measured the staining levels of VEGF-A per unit area by using NIS-Elements (Nikon) and Image-J (NIH). Six mice were analyzed for each genotype and representative images are shown.

It appeared islet capillaries were increased in TG mice based on observations from hematoxylin and eosin staining of pancreatic sections (Fig. 4A, top panels). We previously reported that PECAM1 (a marker for vascular endothelial cells) positive cells were increased in TG islets [15], and that was reconfirmed in the present study (Fig. 4A, middle panels). Interestingly, the immunoreactivity with anti-vascular endothelial growth factor-A (VEGF-A) was increased in islets of TG mice compared to control mice (Fig. 4A, bottom panels). We showed VEGF-A positive cells were β cells, but not α cells, by double staining VEGF-A with insulin or glucagon (Fig. 5). To quantify these results, we performed real-time RT-PCR and densitometry analysis, and showed that both PECAM1 and VEGF-A were significantly increased in TG islets compared to control islets (Fig. 4B and 5B), despite that the number of β cells were decreased in TG islets (Fig. 2A and B). These results indicated that forced expression of constitutively active FoxO1 increased VEGF-A expression in β cells and, thereby, increased the number of capillaries in islets.

### FoxO1 regulates VEGF-A transcription in β cells

We next investigated the mechanism by which FoxO1 regulates VEGF-A expression in β cells. First we tested whether overexpression of constitutively active FoxO1 (FoxO1-ADA) increases VEGF-A expression in cell culture. We infected β TC3 cells or MIN6 cells, two conventional cell lines for pancreatic β cells, with adenovirus expressing FoxO1-ADA. Real-time RT-PCR analyses revealed that VEGF-A mRNA levels were significantly increased by FoxO1-ADA in a dose dependent manner in both cell lines (Fig. 4C). We then constructed a luciferase vector, which contained a mouse VEGF-A promoter (−1217 to +180) and measured the promoter activities. As we expected, the adenovirus mediated expression of FoxO1-ADA increased VEGF-A promoter activities in a dose dependent manner in MIN6 cells (Fig. 4D). Mouse VEGF-A promoter region contains three forkhead responsive elements (FHREs) as indicated in Fig.4E. By using the primers spanning these three FHREs, we performed ChIP assays, which clearly demonstrated that FoxO1 binds to the VEGF-A promoter in intact chromatin (Fig. 4F). Because FoxO3a, another isoform of FoxO family, is also expressed in adult pancreas [10], [22], we tested whether FoxO3a also binds to the VEGF-A promoter in β cells. However, ChIP assays indicated no binding of FoxO3a to the VEGF-A promoter region including three forkhead responsive elements (Fig. 4F). These results indicate that FoxO1 specifically regulates VEGF-A transcription in β cells.

### Islet vascularity and VEGF-A expression in diabetic model mice

**Figure 6:**
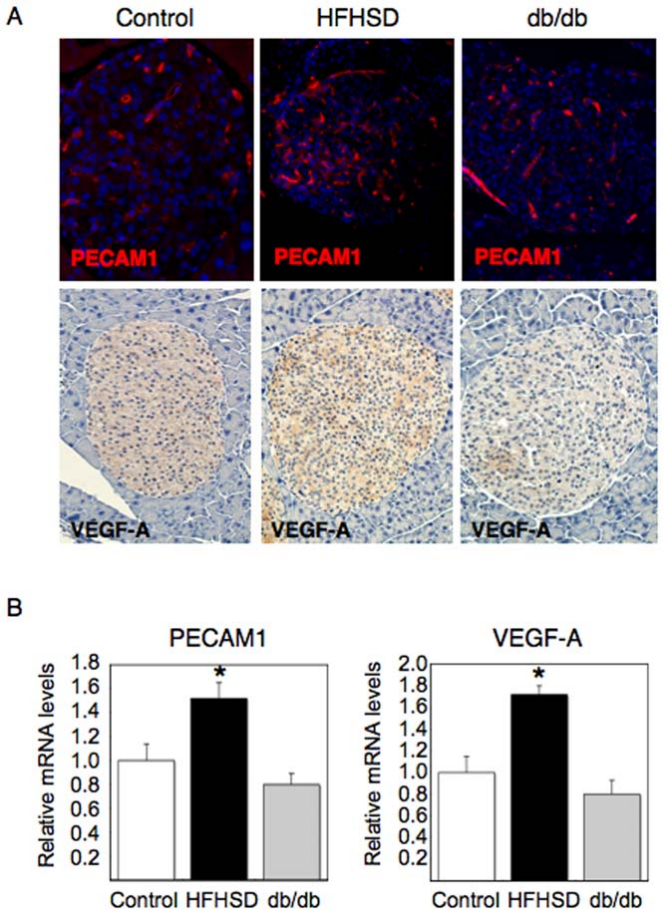
Islet vascularity and VEGF-A expression in HFHSD fed mice and *db/db* mice. (A) Immunohistochemistry with anti-PECAM1 or anti-VEGF-A antibodies in pancreatic sections from HFHSD fed mice, *db/db*, and control mice. Six male mice from each genotype and six sections per mouse were analyzed. Representative images are shown. (B) Islets were isolated from HFHSD fed mice, *db/db*, and control mice and used for analyses by real-time RT-PCR to quantify PECAM1 and VEGF-A mRNA levels. Six male mice were analyzed for each genotype. The results were normalized using GAPDH. Data represent the mean ± SEM fold-increase relative to control mice. An asterisk indicates *P*<0.05 by ANOVA.

Genetic studies have revealed that islet vascular formation, which is regulated by VEGF-A in β cells, is important for the control of blood glucose levels [23], [24], [25]. However, the physiological relevance between VEGF-A/islet vascularity and compensatory β cell hyperplasia against peripheral insulin resistance is still unclear. We investigated the expression levels of PECAM1 and VEGF-A in islets of two animal models for insulin resistance, HFHSD fed mice and *db/db* mice. Interestingly, both PECAM1 and VEGF-A expression in islets were increased in HFHSD fed mice; however, expression was unchanged or tended to be decreased in *db/db* mice (Fig. 6A and 6B). Although both animal models are known to exhibit compensatory β cell hyperplasia, blood glucose levels in ad-lib fed HFHSD mice were normal (121±20 mg/dl); whereas, the levels were considerably high in *db/db* mice (362±38 mg/dl). These results suggested VEGF-A expression levels and islet vascularity may correlate with the compensatory efficiency of β cell hyperplasia. It is of great importance to clarify the physiological relevance of FoxO1 in the regulation of islet vasculature and compensatory β cell hyperplasia in future studies.

## Discussion

In a series of our previous study, we hypothesized that FoxO1 is a negative regulator of β cell proliferation and neogenesis [10], [15]. On the other hand, it has been known that in type2 diabetes, β cell failure is caused by the insulin resistance in β cell per se [1], [2], [3], [4]. In the state of insulin resistance, FoxO1 should be dephosphorylated and activated in β cells. In this study we investigated TG mice expressing active FoxO1 specifically in pancreas and showed that TG mice exhibit hyperglycemia. This report provide *in vivo* evidence that dysregulation of FoxO1 in pancreas causes decreased β cell mass and impaired insulin secretion, leading to the development of diabetes. Our finding that Pdx1 expression is decreased in β cells of TG mice (Fig. 2C) is consistent with our previous hypothesis that FoxO1 inhibits Pdx1 transcription [10]. However, the decrease of MafA expression in β cells of TG mice (Fig. 2D) is inconsistent with our previous hypothesis that FoxO1 induces MafA expression and protects β cells against oxidative stress [21]. The decrease in MafA expression may be accounted for by the reduction of Pdx1, which is a master regulator of MafA transcription.

Another conclusion in our previous study was that FoxO1 regulates cellular differentiation cooperatively with Notch signaling [16]. Consistent with this conclusion, the phenotype of pancreatic morphology in TG mice includes essentially the same abnormalities of pancreas development seen in pancreas specific, constitutively active Notch1 knockin mice, i.e. decrease in exocrine acini, disruption of islet composition, and enlarged pancreatic ducts [17]. The metabolic phenotype could not been analyzed in Notch1 knockin mice because of perinatal lethality. In contrast, we analyzed the metabolic phenotype in adult TG mice and showed TG mice have impaired glucose tolerance and some develop overt diabetes (Fig. 1).

We previously generated the transgenic mice expressing a constitutively active FoxO1 driven by the Ttr promoter (line 305) [26], in which constitutively active FoxO1 is expressed in both liver and pancreatic β cells. Line 305 mice developed diabetes due to increased hepatic glucose production and impaired β cell compensation. The difference between line 305 mice and the TG mice in the present study is that the former exhibited insufficient β cell compensation against hepatic insulin resistance, by contrast, the latter showed significantly impaired insulin secretion and decreased β cell mass.

Despite that β cell mass was reduced in both male and female TG mice, impairment of glucose tolerance was much milder in female TG mice than male TG mice (Fig. 1). Glucose responsive insulin secretion was also impaired in only male, not female, TG mice (Fig. 1B). We speculate that sex hormones may differentially regulate FoxO1 activation or differentially affect β cell function between male and female TG mice; further studies will be needed to elucidate this. It is also of interest that there was a wide variation in blood glucose levels in male TG mice (Fig. 1A and 1G). Therefore, we tested whether the blood glucose level correlates with Pdx1 and/or FoxO1 expression level in TG mice. The results indicated there was a trend for negative correlation between Pdx1 and blood glucose level (R = −0.37) without statistic significance (P = 0.41) and no correlation between FoxO1 and hyperglycemia (data not shown). Thus, we still don't know the reason for a wide variation in blood glucose levels in TG mice. Although there is no statistic significance (P value is 0.08 by ANOVA), the absolute number of α cells tends to be increased (Fig. 2B). These results are consistent with our previous observation that overexression of FoxO1-ADA leads to the differentiation of pancreatic duct cells into α cells [15]. Furthermore, the higher levels of plasma glucagon in TG mice than control mice (Fig. 1E) are likely accounted by the increased α cell number. Although plasma glucagon levels should be logically decreased after feeding, our results demonstrated that plasma glucagon levels were increased in the fed state compared to the fasted state in both TG and control mice (Fig. 1E). This may be due to the difference of the nutrient composition of chow diet (60% carbohydrate, 15% fat and 25% protein) used in this study. It is notable that similar observation (increasing plasma glucagon after feeding) has been reported by other group [27].

Though still controversial, pancreatic duct cells are known to be progenitor for β cells [28]. However, all duct cells are not progenitors, but only a few duct cells develop to new islets. On the other hand, we previously reported that FoxO1 essentially inhibits β cell neogenesis from duct cells, as the loss of FoxO1 in pancreatic duct cells enhanced β cell neogenesis [15]. Therefore, FoxO1 overexpression in pancreatic duct cells increases duct cell proliferation, which leads to pancreatic cyst formation, but at the same time it also inhibits β cell neogenesis from duct progenitors, resulting in the loss of β cells and hyperglycemia.

Cystic disease of the pancreas is classified into two groups, pancreatic cyst and pseudocyst [29]. Pancreatic pseudocysts often follow chronic or acute pancreatitis or blunt trauma to the abdomen. On the other hand, the cause of pancreatic cysts is unknown, except for rare genetic disorders, such as von Hippel-Lindau Disease [30]. There is no report indicating the relevance between FoxO1 and human pancreatic cystic disease, and also no mechanistic implications for FoxO1 regulating pancreatic cyst formation. As we previously reported [10], there are a few Pdx1-positive ductal cells in the adult pancreas. FoxO1-ADA expression in those ductal cells can be speculated to cause cyst formation in the pancreas of TG mice. Therefore, more precise analyses of pancreatic cysts in TG mice may provide novel mechanistic implications for the etiology of these cysts. Furthermore, the association between pancreatic cystic disease and diabetes hasn't been reported. Therefore, it is intriguing that FoxO1 dysregulation in pancreatic cells causes both diabetes and pancreatic polycysts in TG mice. Further investigation will be needed to clarify the association of diabetes and pancreatic cystic disease.

Genetic studies have revealed that islet vascular formation, which is regulated by VEGF-A expression in β cells, is important for the control of blood glucose levels [23], [24], [25]. In this study, we showed FoxO1 regulates VEGF-A expression in β cells and thereby increases capillaries in islets. FoxO1 regulation of VEGF-A/islet vascular formation may contribute to compensatory β cell hyperplasia against peripheral insulin resistance, a hallmark of type 2 diabetes. It seems contradictory that FoxO1 increases islet vascularities while decreasing the number of β cells. However, FoxO1 may function as a double-edged sword, e.g. we previously reported that FoxO1 protects against β cell failure under oxidative stress [21], despite that FoxO1 has been known to inhibit β cell proliferation and neogenesis [10], [15]. Alternative explanation is that FoxO1 overexpression in pancreas indeed reduces β cells and causes hyperglycemia in TG mice, but at the same time FoxO1 increases islet vascularities, which could account for the milder reduction of insulin secretion (Fig. 1E) and the milder glucose intolerance (Fig. 1C), in spite of the severe reduction of β cells (Fig. 2A and 2B).

Very recently Tsunekawa et al. reported that FoxO3a but not FoxO1 regulates IRS2 gene transcription in β cells, despite that both FoxO3a and FoxO1 were capable to bind to the IRS2 promoter [22]. In the present study, we showed only FoxO1, not FoxO3a, recruits to the VEGF-A promoter, providing important implications for target specificity between FoxO1 and FoxO3a in β cells. Therefore, it is intriguing to test in future study how FoxO1 and FoxO3a share a role in the regulation of β cell growth and function.

Although both animal models had peripheral insulin resistance, HFHSD fed mice were resistant to developing hyperglycemia due to effective compensatory β cell hyperplasia, which is associated with increased VEGF-A expression and islet vasculature; whereas, *db/db* mice developed severe diabetes due to insufficient β cell hyperplasia which is accounted for by unchanged or decreased VEGF-A expression and islet vasculature (Fig. 6). In *db/db* mice, it is still unclear whether the lack of leptin signaling correlates with the decreased VEGF-A expression [31]. Furthermore, it remains unknown whether FoxO1 levels in the pancreas are relevant to the difference in these two animal models. Future studies will clarify the physiological relevance between FoxO1 and compensatory β cell hyperplasia against insulin resistance.

In conclusion, we demonstrated that FoxO1 gain of function in the pancreas causes diabetes, polycystic pancreas, and islet hypervascularization. We propose that dysregulation of FoxO1 activity in the pancreas may account for the development of diabetes and pancreatic cysts.
